# On the use of orientation filters for 3D reconstruction in event-driven stereo vision

**DOI:** 10.3389/fnins.2014.00048

**Published:** 2014-03-31

**Authors:** Luis A. Camuñas-Mesa, Teresa Serrano-Gotarredona, Sio H. Ieng, Ryad B. Benosman, Bernabe Linares-Barranco

**Affiliations:** ^1^Instituto de Microelectrónica de Sevilla (IMSE-CNM), CSIC y Universidad de SevillaSevilla, Spain; ^2^UMR_S968 Inserm/UPMC/CNRS 7210, Institut de la Vision, Université de Pierre et Marie CurieParis, France

**Keywords:** stereovision, neuromorphic vision, Address Event Representation (AER), event-driven processing, convolutions, gabor filters

## Abstract

The recently developed Dynamic Vision Sensors (DVS) sense visual information asynchronously and code it into trains of events with sub-micro second temporal resolution. This high temporal precision makes the output of these sensors especially suited for dynamic 3D visual reconstruction, by matching corresponding events generated by two different sensors in a stereo setup. This paper explores the use of Gabor filters to extract information about the orientation of the object edges that produce the events, therefore increasing the number of constraints applied to the matching algorithm. This strategy provides more reliably matched pairs of events, improving the final 3D reconstruction.

## Introduction

Biological vision systems are known to outperform any modern artificial vision technology. Traditional frame-based systems are based on capturing and processing sequences of still frames. This yields a very high redundant data throughput, imposing high computational demands. This limitation is overcome in bio-inspired event-based vision systems, where visual information is coded and transmitted as events (spikes). This way, much less redundant information is generated and processed, allowing for faster and more energy efficient systems.

Address Event Representation (AER) is a widely used bio-inspired event-driven technology for coding and transmitting (sensory) information (Sivilotti, 1991; Mahowald, 1992; Lazzaro et al., 1993). In AER sensors, each time a pixel senses relevant information (like a change in the relative light) it asynchronously sends an event out, which can be processed by event-based processors (Venier et al., 1997; Choi et al., 2005; Silver et al., 2007; Khan et al., 2008; Camuñas-Mesa et al., 2011, 2012; Zamarreño-Ramos et al., 2013). This way, the most important features pass through all the processing levels very fast, as the only delay is caused by the propagation and computation of events along the processing network. Also, only pixels with relevant information send out events, reducing power and bandwidth consumption. These properties (high speed and low energy) are making AER sensors very popular, and different sensing chips have been reported for vision (Lichtsteiner et al., 2008; Leñero-Bardallo et al., 2010, 2011; Posch et al., 2011; Serrano-Gotarredona and Linares-Barranco, 2013) or auditory systems (Lazzaro et al., 1993; Cauwenberghs et al., 1998; Chan et al., 2007).

The development of Dynamic Vision Sensors (DVS) was very important for high speed applications. These devices can track extremely fast objects with standard lighting conditions, providing an equivalent sampling rate higher than 100 KFrames/s. Exploiting this fine time resolution provides a new mean for achieving stereo vision with fast and efficient algorithms (Rogister et al., 2012).

Stereovision processing is a very complex problem for conventional frame-based strategies, due to the lack of precise timing information as used by the brain to solve such tasks (Meister and Berry II, 1999). Frame-based methods usually process sequentially sets of images independently, searching for several features like orientation (Granlund and Knutsson, 1995), optical flow (Gong, 2006) or descriptors of local luminance (Lowe, 2004). However, event-based systems can compute stereo information much faster using the precise timing information to match pixels between different sensors. Several studies have applied events timing together with additional constraints to compute depth from stereo visual information (Marr and Poggio, 1976; Mahowald and Delbrück, 1989; Tsang and Shi, 2004; Kogler et al., 2009; Domínguez-Morales et al., 2012; Carneiro et al., 2013; Serrano-Gotarredona et al., 2013).

In this paper, we explore different ways to improve 3D object reconstruction using Gabor filters to extract orientation information from the retinas events. For that, we use two DVS sensors with high contrast sensitivity (Serrano-Gotarredona and Linares-Barranco, 2013), whose output is connected to a convolutional network hardware (Zamarreño-Ramos et al., 2013). Different Gabor filter architectures are implemented to reconstruct the 3D shape of objects. In section Neuromorphic Silicon Retina, we describe briefly the DVS sensor used. Section Stereo Calibration describes the calibration method used in this work. In section Event Matching, we detail the matching algorithm applied, while section 3D Reconstruction shows the method for reconstructing the 3D coordinates. Finally, section Results provides experimental results.

## Neuromorphic silicon retina

The DVS used in this work is an AER silicon retina with 128 × 128 pixels and increased contrast sensitivity, allowing the retina to detect contrast as low as 1.5% (Serrano-Gotarredona and Linares-Barranco, 2013). The output of the retina consists of asynchronous AER events that represent a change in the sensed relative light. Each pixel independently detects changes in log intensity larger than a threshold since the last emitted event θ_*ev*_ = |*I*(*t*) − *I*(*t*_last−spike_) |/*I*(*t*).

**Figure 1:**
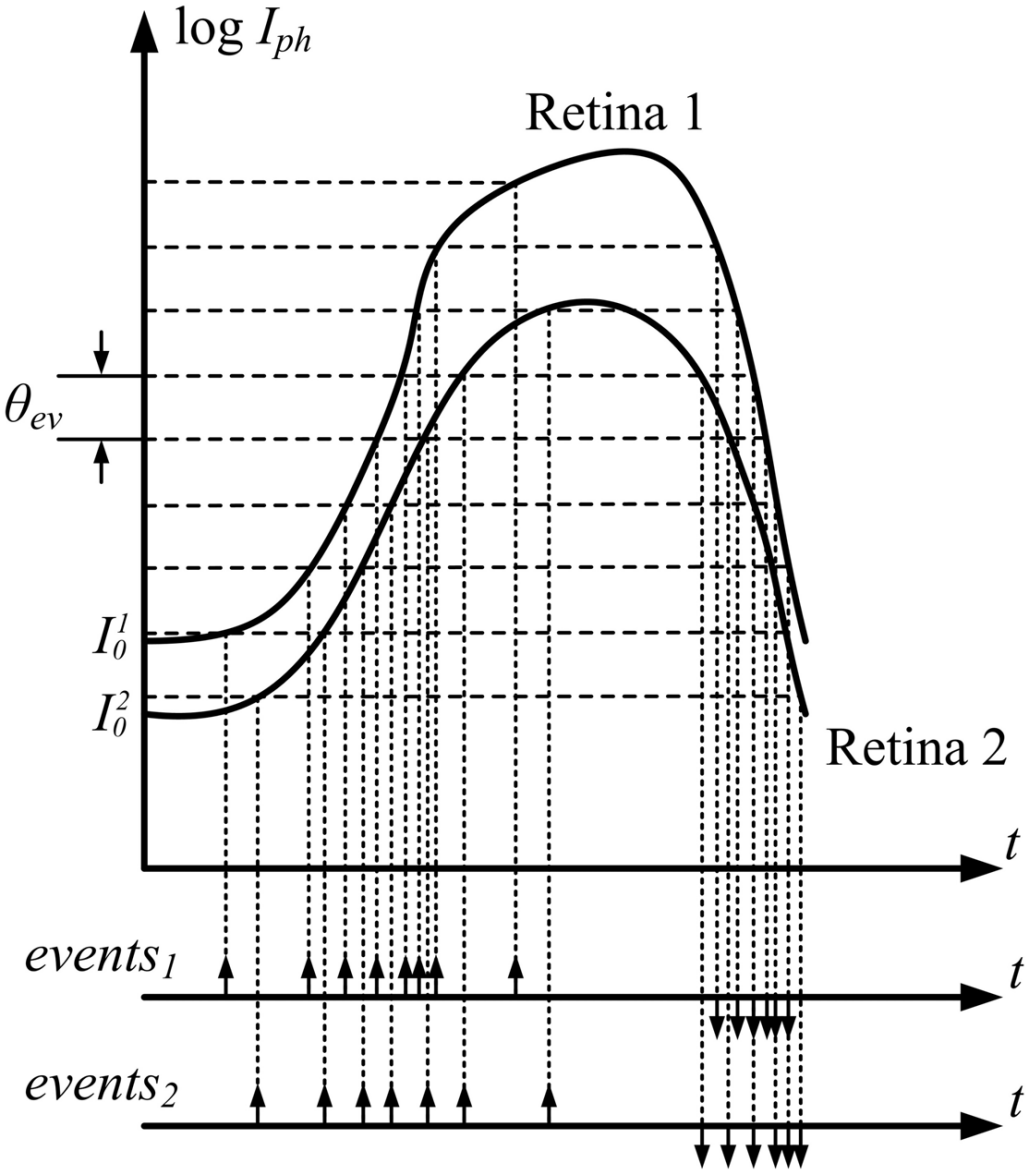
**Data driven asynchronous event generation for two equivalent pixels in Retina 1 and Retina 2**. Because of intra-die pixel mismatch and inter-die sensor mismatch, both response curves differ.

The most important property of these sensors is that pixel information is obtained not synchronously at fixed frame rate δ t, but asynchronously driven by data at fixed relative light increments θ_*ev*_, as shown in Figure 1. This figure represents the photocurrent transduced by two pixels in two different retinas in a stereo setup, configured so that both pixels are sensing an equivalent activity. Even though if both are sensing exactly the same light, the transduced currents are different, given the change in initial conditions (*I*^1^_0_ and *I*^2^_0_) and mismatch between retina pixels that produce a different response to the same stimulus. As a consequence, the trains of events generated by these two pixels are not identical, as represented in Figure 1.

The events generated by the pixels can have either positive or negative polarity, depending on whether the light intensity increased or decreased. These events are transmitted off-chip, timestamped and sent to a computer using a standard USB connection.

## Stereo calibration

Before using a pair of retinas for sensing and matching pairs of corresponding events and reconstruct each event in 3D, both retinas relative positions and orientations need to be calibrated.

Let us use lower case to denote a 2D point in the retina sensing plane as *m* = [*x y*]^*T*^, and capital letter to denote the corresponding 3D point in real space as *M* = [*X Y Z*]^*T*^. Augmented vectors are built by adding 1 as the last element: $\tilde{m}$ = [*x y* 1]^*T*^ and $\tilde{M}$ = [*X Y Z* 1]^*T*^. Under the assumptions of the pinhole camera model, the relationship between $\tilde{m}$ and $\tilde{M}$ is given by Hartley and Zisserman (2003):

(1) $$\tilde{m}=P_{i}\cdot \tilde{M}$$

where *P*_*i*_ is the projection matrix for camera *i*. In order to obtain the projection matrices of a system, many different techniques have been proposed, and they can be classified into the following two categories (Zhang, 2000):

- Photogrammetric calibration: using a calibration object with known geometry in 3D space. This calibration object usually consists of two or three planes orthogonal to each other (Faugeras, 1993).
- Self-calibration: the calibration is implemented by moving the cameras in a static scene obtaining several views, without using any calibration object (Maybank and Faugeras, 1992).

**Figure 2:**
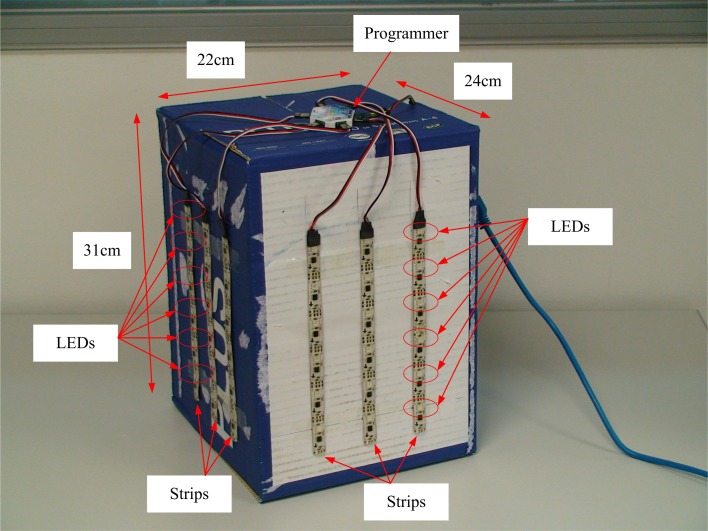
**Photograph of the calibration structure, with 36 LEDs distributed in two orthogonal planes**. The size of the object is shown in the figure.

In this work, we have implemented a calibration technique based on a known 3D object, consisting of 36 points distributed in two orthogonal planes. Using this fixed pattern, we calibrate two DVS. A blinking LED was placed in each one of these 36 points. LEDs blinked sequentially one at a time, producing trains of spikes in several pixels at both sensors. From these trains of spikes, we needed to extract the 2D calibration coordinates $\tilde{m}$^*j*^_*i*_, where *i* = 1, 2 represents each silicon retina and *j* = 1,… 36 represents the calibration points (see Figure 2). There are two different approaches to obtain these coordinates: with pixel or sub-pixel resolution. In the first one, we decided that the corresponding 2D coordinate for a single LED was represented by the pixel which responded with a higher firing rate. In the second one, we selected a small cluster of pixels which responded to that LED with a firing rate above a certain threshold, and we calculated the average coordinate, obtaining sub-pixel accuracy.

After calculating $\tilde{m}$^*j*^_1_ and $\tilde{m}$^*j*^_2_ (*j* = 1,… 36) and knowing $\tilde{M}$^*j*^, we can apply any algorithm that was developed for traditional frame-based computer vision (Longuet-Higgins, 1981) to extract *P*_1_ and *P*_2_ (Hartley and Zisserman, 2003). More details can be found in Calculation of Projection Matrix P in Supplementary Material.

The fundamental matrix *F* relates the corresponding points obtained from two cameras, and is defined by the equation:

(2) $${\tilde{m}}_{1}^{T}F{\tilde{m}}_{2}\;=\;0$$

where $\tilde{m}$_1_ and $\tilde{m}$_2_ are a pair of correspondent 2D points in both cameras (Luong, 1992). This system can be solved using the 36 pairs of points mentioned before (Benosman et al., 2011).

## Event matching

In stereo vision systems, a 3D point in space *M* is projected onto the focal planes of both cameras in pixels *m*_1_ and *m*_2_, therefore generating events *e*(*m*^*i*^_1_, *t*) and *e*(*m*^*i*^_2_, *t*). Reconstructing the original 3D point requires matching each pair of events produced by point *M* at time *t* (Carneiro et al., 2013). For that, we implemented two different matching algorithms (A and B) based on a list of restrictions applied to each event in order to find its matching pair. These algorithms are described in the following subsections.

### Retinas events matching algorithm (A)

This first algorithm (Carneiro et al., 2013) consists of applying the following restrictions (1–4) to the events generated by the silicon retinas. Therefore, for each event generated by retina 1 we have to find out how many events from retina 2 satisfy the 4 restrictions. If the answer is only one single event, it can be considered its matching pair. Otherwise, it is not possible to determine the corresponding event, and it will be discarded.

#### Restriction 1: temporal match

One of the most useful advantages of event-driven DVS based vision sensing and processing is the high temporal resolution down to fractions of micro seconds (Lichtsteiner et al., 2008; Posch et al., 2011; Serrano-Gotarredona and Linares-Barranco, 2013). Thus, in theory, two identical DVS cameras observing the same scene should produce corresponding events simultaneously (Rogister et al., 2012). However, in practice, there are many non-ideal effects that end up introducing appreciable time differences (up to many milli seconds) between corresponding events:

- inter-pixel and inter-sensor variability in the light-dependent latency since a luminance change is sensed by the photodiode until it is amplified, processed and communicated out of the chip;
- presence of noise at various stages of the circuitry;
- variability in inter-pixel and inter-sensor contrast sensitivity; and
- randomness of pixel initial conditions when a change of light happens.

**Figure 3:**
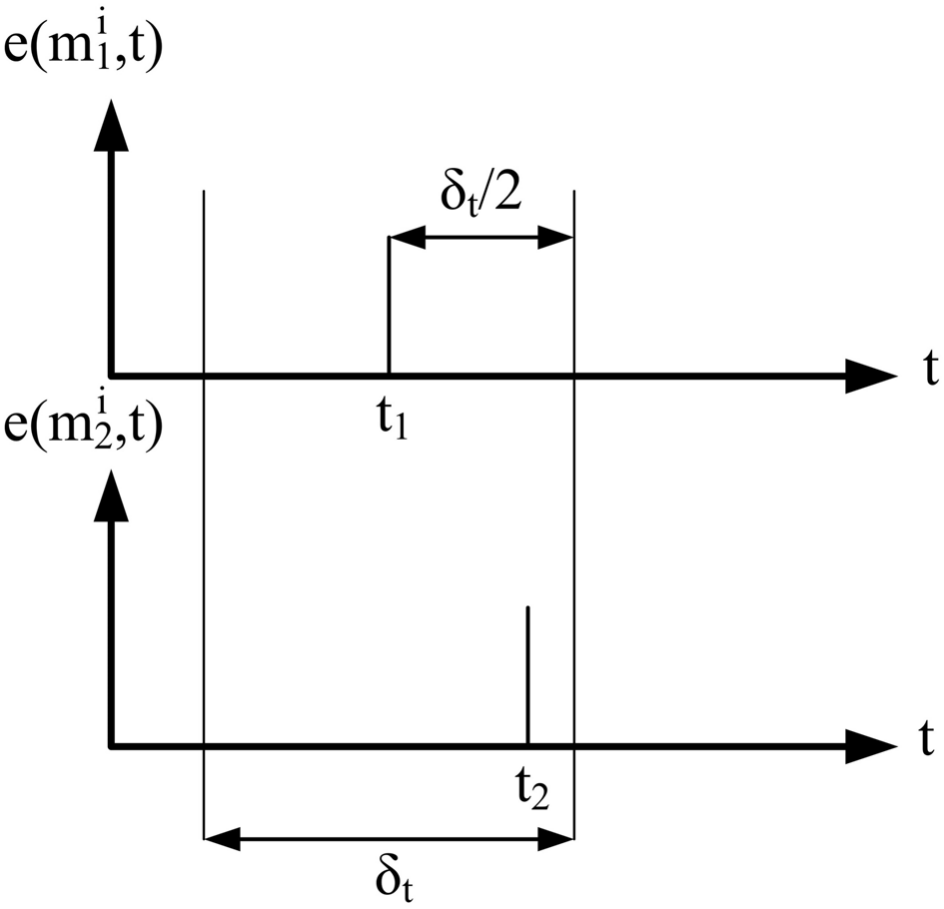
**Temporal match**. Two events can be considered as candidates to match if they are generated within a certain time interval δ_*t*_.

Nonetheless, corresponding events occur within a milli second range time window, depending on ambient light (the lower light, the wider the time window). As a consequence, this first restriction implies that for an event *e*(*m*^*i*^_1_, *t*_1_), only those events *e*(*m*^*i*^_2_, *t*_2_) with |*t*_1_ − *t*_2_| < δ_*t*_/2 can be candidates to match, as shown in Figure 3. In our experimental setup we used a value of δ _*t*_ = 4 ms, which gave the best possible result under standard interior lighting conditions.

#### Restriction 2: epipolar restriction

As is described in detail in (Hartley and Zisserman, 2003), when a 3D point in space *M* is projected onto pixel *m*_1_ in retina 1, the corresponding pixel *m*_2_ lies on an epipolar line in retina 2 (Carneiro et al., 2013). Using this property, a second restriction is added to the matching algorithm using the fundamental matrix *F* to calculate the epipolar line *Ep*_2_ in retina 2 corresponding to event *m*_1_ in retina 1 (*Ep*_2_ (*m*_1_) = *F*^*T*^$\tilde{m}$_1_). Therefore, only those events *e*(*m*^*i*^_2_, *t*_2_) whose distance to *Ep*_2_ is less than a given limit δ_*Ep*__*i*_ can be candidates to match. In our experiments we used a value of δ_*Ep*__*i*_ = 1 *pixel*.

#### Restriction 3: ordering constraint

For a practical stereo configuration of retinas where the angle between their orientations is small enough, a certain geometrical constraint can be applied to each pair of corresponding events. In general, the horizontal coordinate of the events generated by a retina is always larger than the horizontal coordinate of the corresponding events generated by the other retina.

#### Restriction 4: polarity

The silicon retinas used in our experimental setup generate output events when they detect a change in luminance in a pixel, indicating in the polarity of the event if that change means increasing or decreasing luminance (Lichtsteiner et al., 2008; Posch et al., 2011; Serrano-Gotarredona and Linares-Barranco, 2013). Using the polarity of events, we can impose the condition that two corresponding events in both retinas must have the same polarity.

### Gabor filter events matching algorithm (B)

We propose a new algorithm where we use the orientation of the object edges to improve the matching, increasing the number of correctly matched events.

If the focal planes of two retinas in a stereo vision system are roughly vertically aligned and have a small horizontal vergence, the orientation of observed edges will be approximately equal provided that the object is not too close to the retinas. A static DVS produces events when observing moving objects, or more precisely, when observing the edges of moving objects. Therefore, correspondent events in the two retinas are produced by the same moving edges, and consequently the observed orientation of the edge should be similar in both retinas. An edge would appear with a different angle in both retinas only when it is relatively close to them, and in practice this does not happen because of two reasons^1^:

1. Since both cameras have small horizontal vergence, the object would be out of the overlapping field of view of the 2 retinas far before being so close. In that case, we do not have stereo vision anymore.
2. The minimal focusing distance of the cameras' lenses limits the maximal vergence.

Considering that, we can assume that the orientation of an edge will be approximately the same in both retinas under our working conditions. Under different conditions, an epipolar rectification should be applied to the stereo system to ensure the orientations of the edges to be identical in the two cameras. This operation consists in estimating the homographies mapping and scaling the events of each retina into two focal planes parallel to the stereo baseline (Loop and Zhang, 1999). Lines in the rectified focal planes are precisely the epipolar lines of the stereo system. This rectification should be carried out at the same time than the retinas calibration.

**Figure 4:**
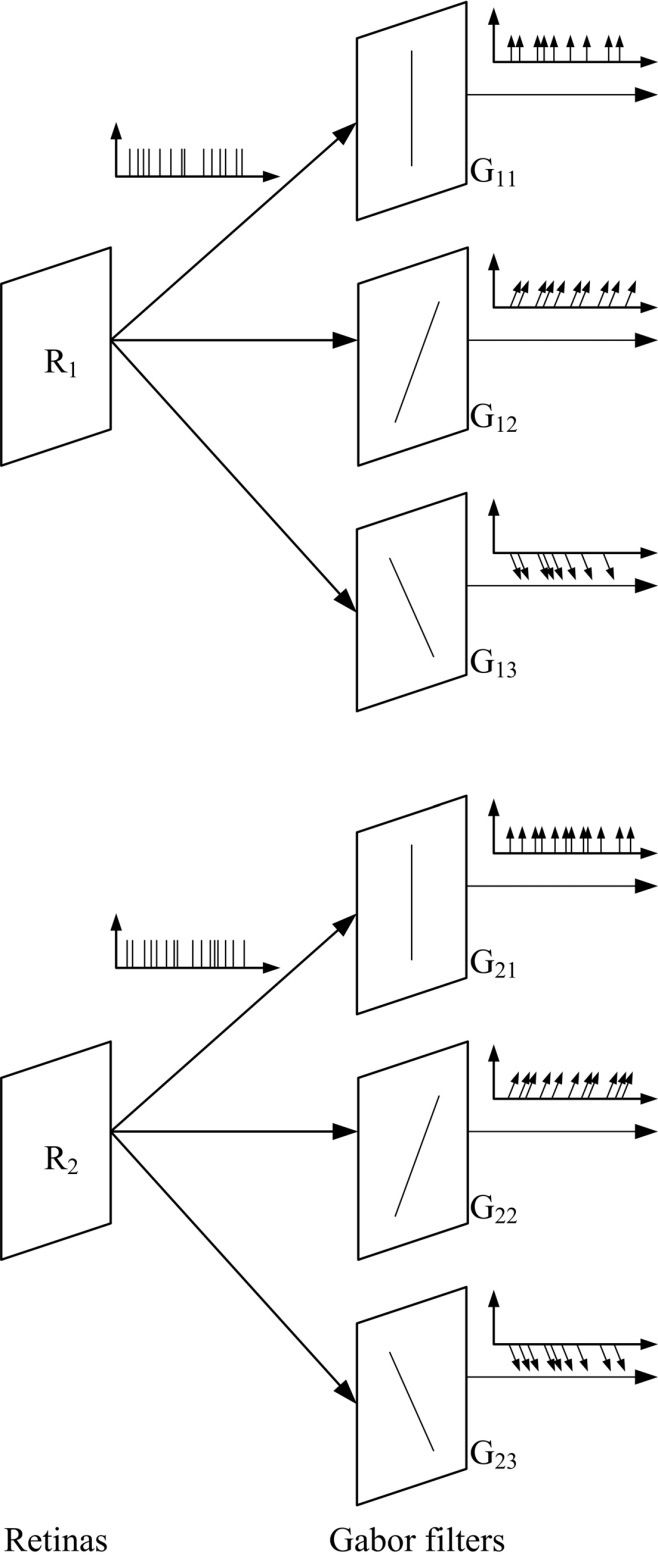
**Illustration of the use of 3 Gabor filters with different orientations to the output of both retinas**. The events generated by the filters carry additional information, as they represent the orientation of the edges.

The application of banks of Gabor filters to the events generated by both retinas provides information about the orientation of the object edges that produce the events as shown in Figure 4. This way, by using Gabor filters with different angles we can apply the previously described matching algorithm to pairs of Gabor filters with the same orientation. Thus, the new matching algorithm is as follows. The events coming out of retinas *R*_1_ and *R*_2_ are processed by Gabor filters *G*_1*x*_ and *G*_2*x*_, respectively (with *x* = 1, 2, … *N*, being *N* the number of orientation filters for each retina). Then, for each pair of Gabor filters *G*_1*x*_ and *G*_2*x*_, conditions 1–4 are applied to obtain matched events for each orientation. Therefore, the final list of matched events will be obtained as the union of all the lists of matched events obtained for each orientation.

## 3D reconstruction

The result provided by the previously described matching algorithm is a train of pairs of corresponding events. Each pair consists of two events with coordinates *m*_1_ = (*x*_1_,*y*_1_)^*T*^ and *m*_2_ = (*x*_2_,*y*_2_)^*T*^. The relationship between $\tilde{m}$ and $\tilde{M}$ for both retinas is given by:

(3) $$\begin{array}{l} {\tilde{m}}_{1}\times P_{1}\tilde{M}=0\\ {\tilde{m}}_{2}\times P_{2}\tilde{M}=0 \end{array}$$

where *P*_1_ and *P*_2_ represent the projection matrices calculated during calibration, and $\tilde{M}$ is the augmented vector corresponding to the 3D coordinate that must be obtained. These equations can be solved as a linear least squares minimization problem (Hartley and Zisserman, 2003), giving the final 3D coordinates *M* = [*X Y Z*]^*T*^ as a solution. More details can be found in Calculation of Reconstructed 3D Coordinates in Supplementary Material.

## Results

In this Section, we describe briefly the hardware setup used for the experiments, then we show a comparison between the different calibration methods, after that we characterize the 3D reconstruction method, and finally we present results on the reconstruction of 3D objects.

### Hardware setup

**Figure 5:**
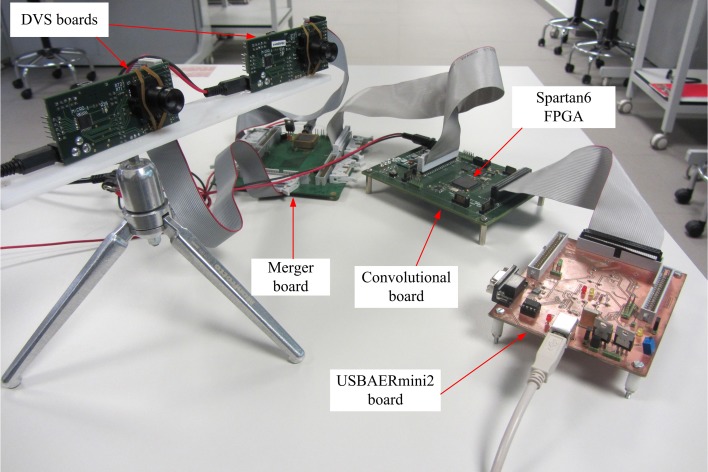
**Experimental stereo setup**.

The event-based stereo vision processing has been tested using two DVS sensor chips (Serrano-Gotarredona and Linares-Barranco, 2013) whose outputs are connected to a merger board (Serrano-Gotarredona et al., 2009) which sends the events to a 2D grid array of event-based convolution modules implemented within a Spartan6 FPGA. This scheme has been adapted from a previous one that used a Virtex6 (Zamarreño-Ramos et al., 2013). The Spartan6 was programmed to perform real-time edge extraction on the visual flow from the retinas. Finally, a USBAERmini2 board (Serrano-Gotarredona et al., 2009) was used to timestamp all the events coming out of the Spartan6 board and send them to a computer through a high-speed USB2.0 port (see Figure 5).

**Figure 6:**
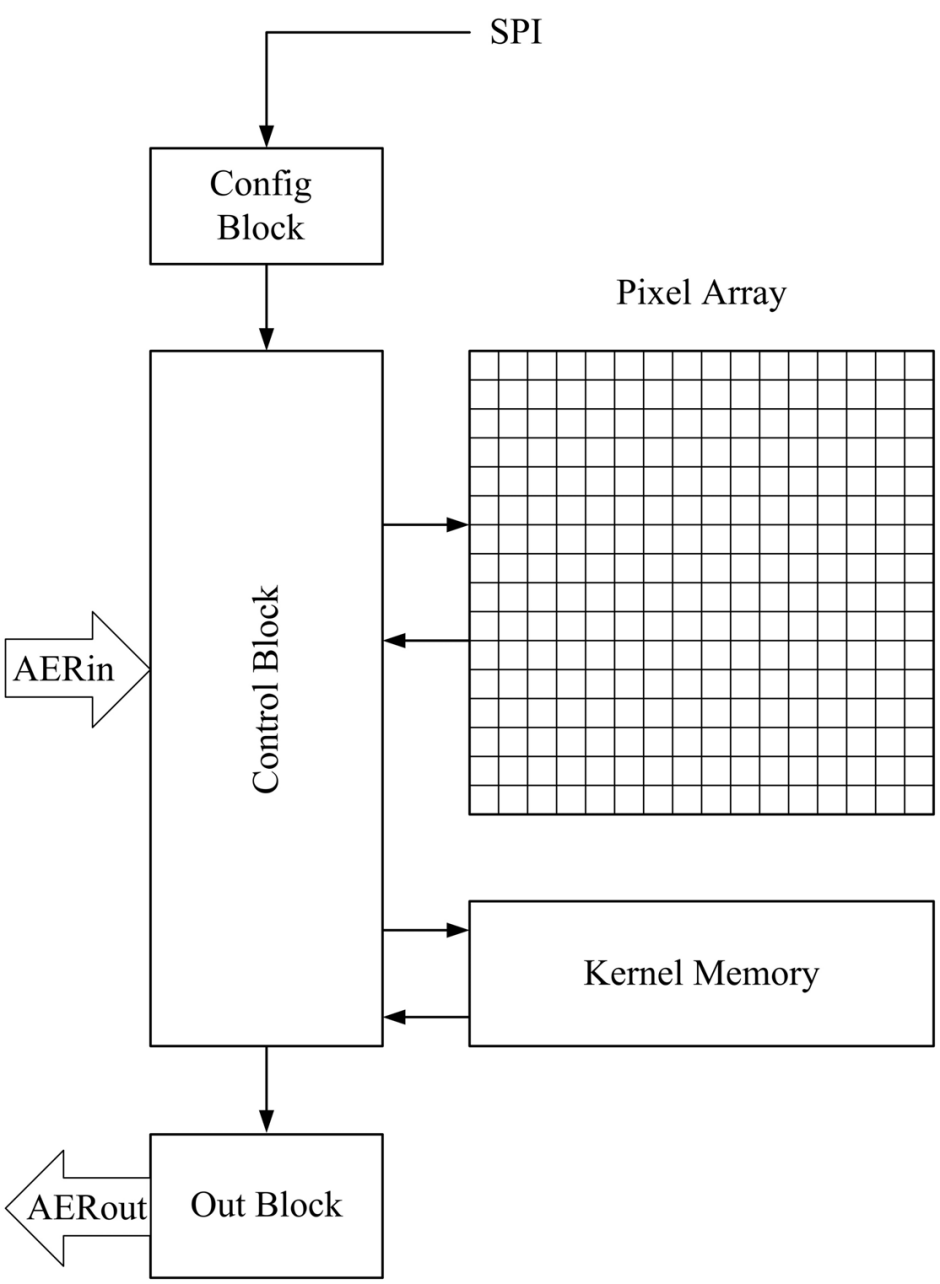
**Block diagram for the convolutional block implemented on FPGA**.

The implementation of each convolution module in the FPGA is represented in Figure 6. It consists of two memory blocks (one to store the pixel values, and the other to store the kernel), a control block that performs the operations, a configuration block that receives all the programmable parameters, and an output block that sends out the events. When an input event arrives, it is received by the control block, which implements the handshaking and calculates which memory positions must be affected by the operation. In particular, it must add the kernel values to the pixels belonging to the appropriate neighborhood around the address of the input event, as done in previous event-driven convolution processors (Serrano-Gotarredona et al., 1999, 2006, 2008, 2009; Camuñas-Mesa et al., 2011, 2012). At the same time, it checks if any of the updated pixels has reached its positive or negative threshold, in that case resetting the pixel and sending a signed event to the output block. A programmable forgetting process decreases linearly the value of all the pixels periodically, making the pixels behave like leaky integrate-and-fire neurons.

**Figure 7:**
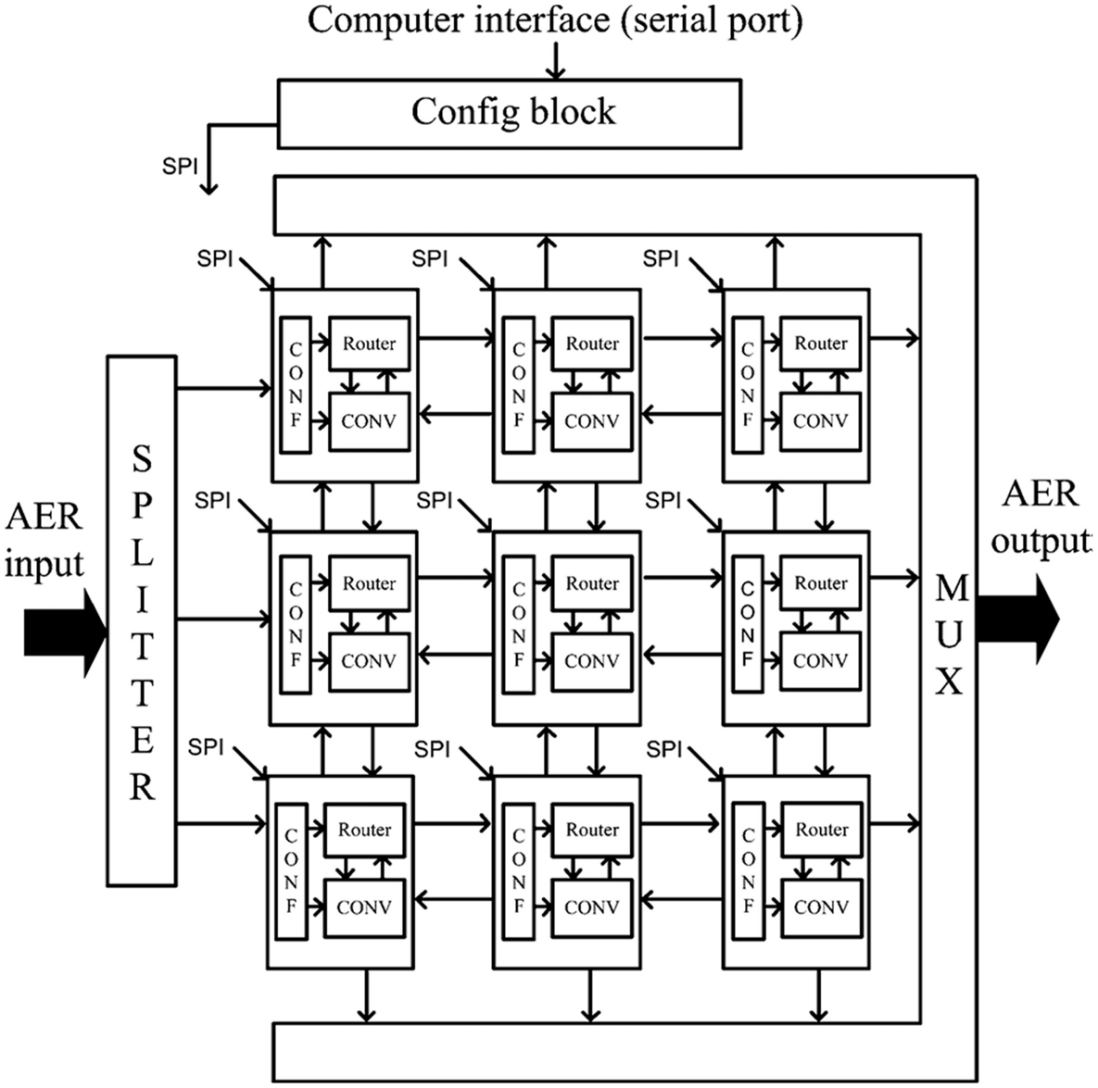
**Block diagram for a sample network with 3 × 3 convolutional blocks implemented on FPGA**.

Several convolutional modules can be arranged in a 2D mesh, each one communicating bidirectionally with all four neighbors, as illustrated in Figure 7 (Zamarreño-Ramos et al., 2013). Each module is characterized by its module coordinate within the array. Address events are augmented by adding either the source or destination module coordinate. Each module includes an AER router which decides how to route the events (Zamarreño-Ramos et al., 2013). This way, any network architecture can be implemented, like the one shown in Figure 4 with any number of Gabor filters. Each convolutional module is programmed to extract a specific orientation by writing the appropriate kernel. In our experiments, the resolution of the convolutional blocks is 128 × 128 pixels.

In order to compensate the mismatch between the two DVS chips, an initial procedure must be implemented. This procedure consists of setting the values of the bias signals which control the sensitivity of the photosensors to obtain approximately the same number of events in response to a fixed stimulus in both retinas.

### Calibration results

In order to calibrate the setup with both DVS retinas (with a baseline distance of 14 cm, being the retinas approximately aligned and the focal length of the lenses 8 mm), we built a structure of 36 blinking LEDs distributed in two orthogonal planes, each with an array of 6 × 3 LEDs with known 3D coordinates in each plane (see Figure 2). The horizontal distance between LEDs is 5 cm, while the vertical separation is 3.5 cm. This structure was placed in front of the DVS stereo setup at approximately 1 m distance, and the events generated by the retinas were recorded by the computer. The LEDs would blink sequentially, so that when one LED produces events no other LED is blinking. This way, during a simultaneous event burst in both cameras, there is only one LED in 3D space blinking, resulting in a unique spatial correspondence between the events produced in both retinas and the original 3D position. This recording was processed offline to obtain the 2D coordinates of the LEDs projected in both retinas following two different approaches:

1. We represent a 2D image coding the number of spikes generated by each pixel. This way for each LED we obtain a cluster of pixels with large values. The coordinate of the pixel with the largest value in each cluster is considered to be the 2D projection of the LED. The accuracy of this measurement is one pixel.
2. Using the same 2D image, the following method is applied. First, all those pixels with a number of spikes below a certain threshold are set to zero, while all those pixels above the threshold are set to one, obtaining a binarization of the image. Figure S1 in Calculation of Projection Matrix P in Supplementary Material shows an example of a 2D binarized image obtained for one DVS, where the 36 clusters represent the responses to the blinking LEDs. Then, for each cluster of pixels we calculate the mean coordinate, obtaining the 2D projection of the LEDs with sub-pixel resolution.

**Figure 8:**
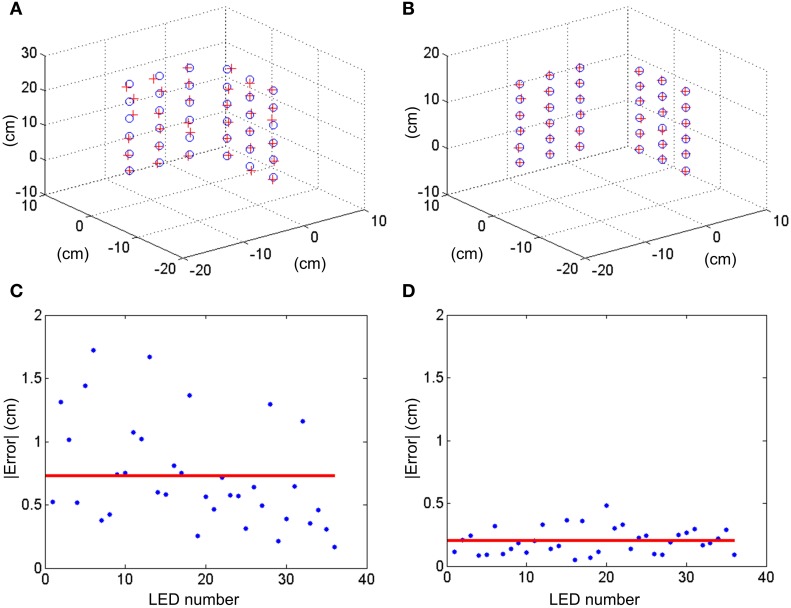
**3D reconstruction of the coordinates of the calibration LEDs**. **(A)** With pixel resolution and **(B)** with sub-pixel resolution. Blue circles represent the real location of the LEDs, while red crosses indicate the reconstructed coordinate. **(C,D)** Show the measured errors absolute value in cm for approaches 1 and 2, respectively. Red lines represent the mean error.

In both cases, these 2D coordinates together with the known 3D positions of the LEDs in space are used to calculate the projection matrices *P*_1_ and *P*_2_, and the fundamental matrix *F* following the methods described in section Stereo Calibration. To validate the calibration, *P*_1_ and *P*_2_ were used to reconstruct the 3D calibration pattern following the method described in section 3D Reconstruction, obtaining the results shown in Figures 8A,B. The reconstruction error is measured as the distance between each original 3D point and its corresponding reconstructed position, giving the results shown in Figures 8C,D. As can be seen in the figure, the mean reconstruction error for approach 1 is 7.3 mm with a standard deviation of 4.1 mm, while for approach 2 it is only 2 mm with a standard deviation of 1 mm. This error is comparable to the size of each LED (1 mm).

### Precision characterization

**Figure 9:**
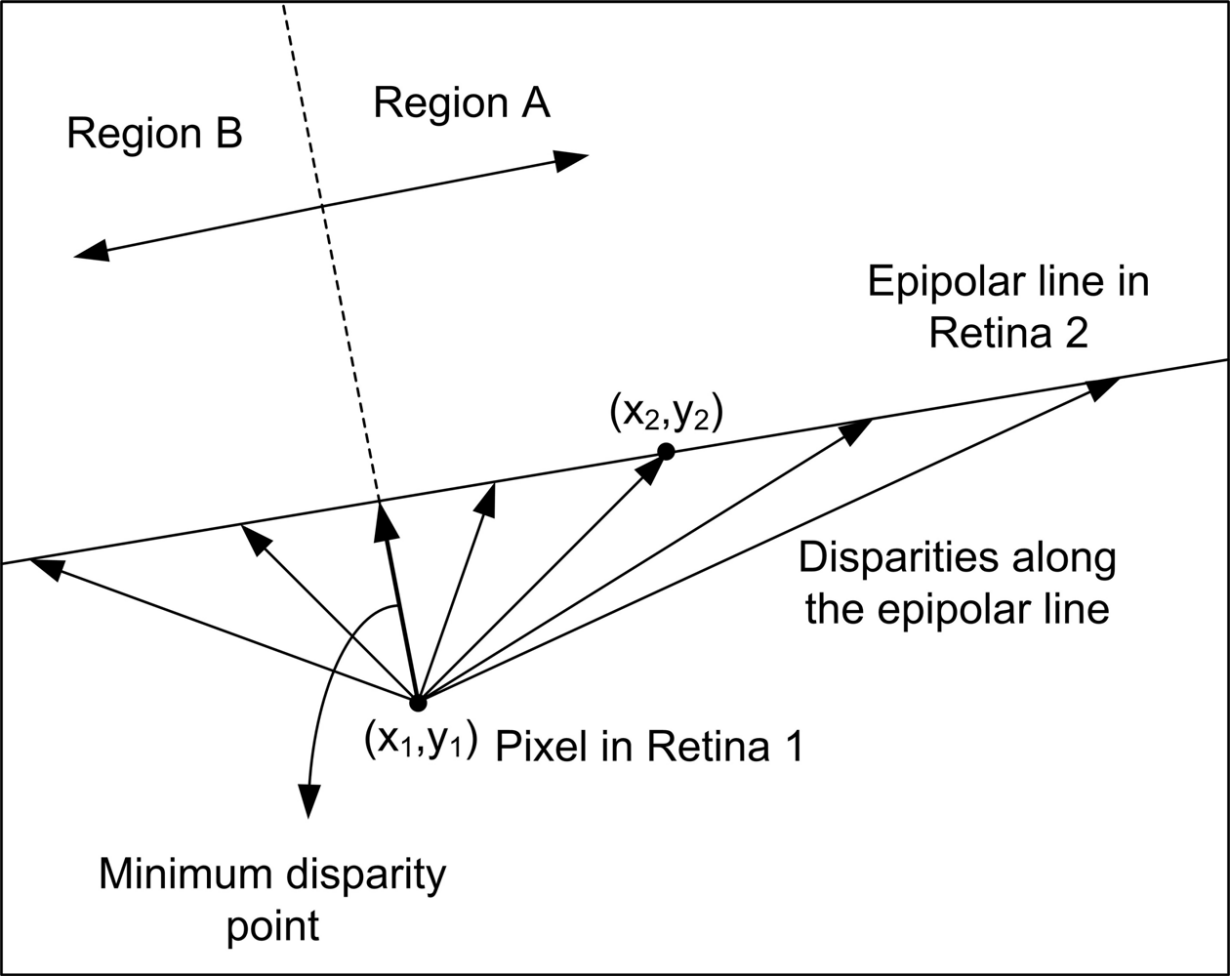
**Measurement of the disparity (distance) between a pixel in Retina 1 and its corresponding epipolar line in Retina 2**. The minimum disparity point separates Region A and B.

**Figure 10:**
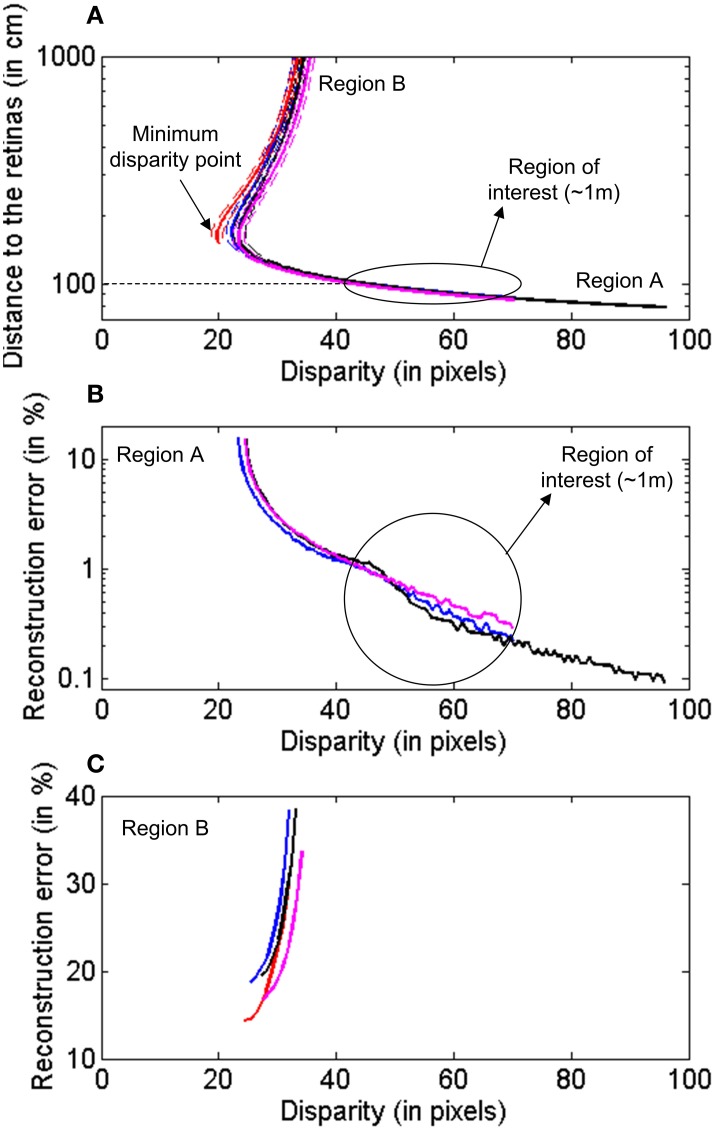
**Characterization of the 3D reconstruction of the epipolar lines for different pixels in Retina 1**. Each color represents a different pixel. **(A)** Distance between the reconstructed points and the retinas for different disparity values. The dashed lines represent the upper and lower limits associated to the allowed deviation around the epipolar line. **(B)** Reconstruction error for 3D points closer to the retinas, Region A. **(C)** Reconstruction error for points farther from the retinas, Region B.

Using the calibration results obtained in the previous subsection, we performed the following evaluation of the 3D reconstruction method. For a fixed pixel *m*^1^_1_ in Retina 1, we used the fundamental matrix *F* to calculate the corresponding epipolar line in Retina 2 *Ep*^1^_2_, as represented in Figure 9. Although a perfect alignment between the two retinas would produce an epipolar line parallel to the x-axis and crossing the pixel position [minimum disparity point coincident with (*x*_1_,*y*_1_)], we represent a more general case, where the alignment is performed manually and is not perfect. This case is illustrated in Figure S1 (see Calculation of Projection Matrix P in Supplementary Material), where we show the 2D images representing the activity recorded by both retinas during calibration. The orientations of the epipolar lines indicate that the alignment is not perfect. The mean disparity for the LEDs coordinates is 24.55 pixels. Considering that we admit a deviation around the epipolar line of δ_*Ep*__*i*_ = 1 *pixel* in the matching algorithm, we calculated two more lines, an upper and a lower limit, given by the distance of ±1 *pixel* to the epipolar line. Using projection matrices *P*_1_ and *P*_2_, we reconstructed the 3D coordinates for all the points in these three lines. We repeated the procedure for a total of four different pixels in Retina 1 *m*^*i*^_1_ (i = 1, 2, 3, 4) distributed around the visual space, obtaining four sets of 3-dimensional lines. In Figure 10A, we represent the distance between these 3D points and the retinas for each disparity value [the disparity measures the 2D euclidean distance between the projections of a 3D point in both retinas (*x*_1_,*y*_1_) and (*x*_2_,*y*_2_)], where each color corresponds to a different pixel *m*^*i*^_1_ in Retina 1, and the dashed lines represent the upper and lower limits given by the tolerance of 1 pixel around the epipolar lines. As can be seen in the figure, each disparity has two different values of distance associated, which represent the two possible points in *Ep*^*i*^_2_ which are at the same distance from *m*^*i*^_1_. This effect results in two different zones in each trace (regions A and B in Figure 9), which correspond to two different regions in the 3D space, where the performance of the reconstruction changes drastically. Therefore, we consider both areas separately in order to estimate the reconstruction error. Using the range of distances given by Figure 10A between each pair of dashed lines, we calculate the reconstruction error for each disparity value as (*d*_*max*_ − *d*_*min*_)/μ _*d*_, where *d*_*max*_ and *d*_*min*_ represent the limits of the range of distance at that point, and μ _*d*_ is the mean value. Figure 10B shows the obtained error for the 3D points located in the closer region (A), while Figure 10C corresponds to the points farther from the retinas (Region B). In both figures, each line represents a different pixel *m*^*i*^_1_ in Retina 1. As shown in Figure 10B, the reconstruction error in the area of interest (around 1m distance from the retinas) is less than 1.5%. Note that the minimum disparity value is around 20 pixels (while a perfect alignment would give 0), showing the robustness of the method for manual approximate alignment.

### 3D reconstruction

**Figure 11:**
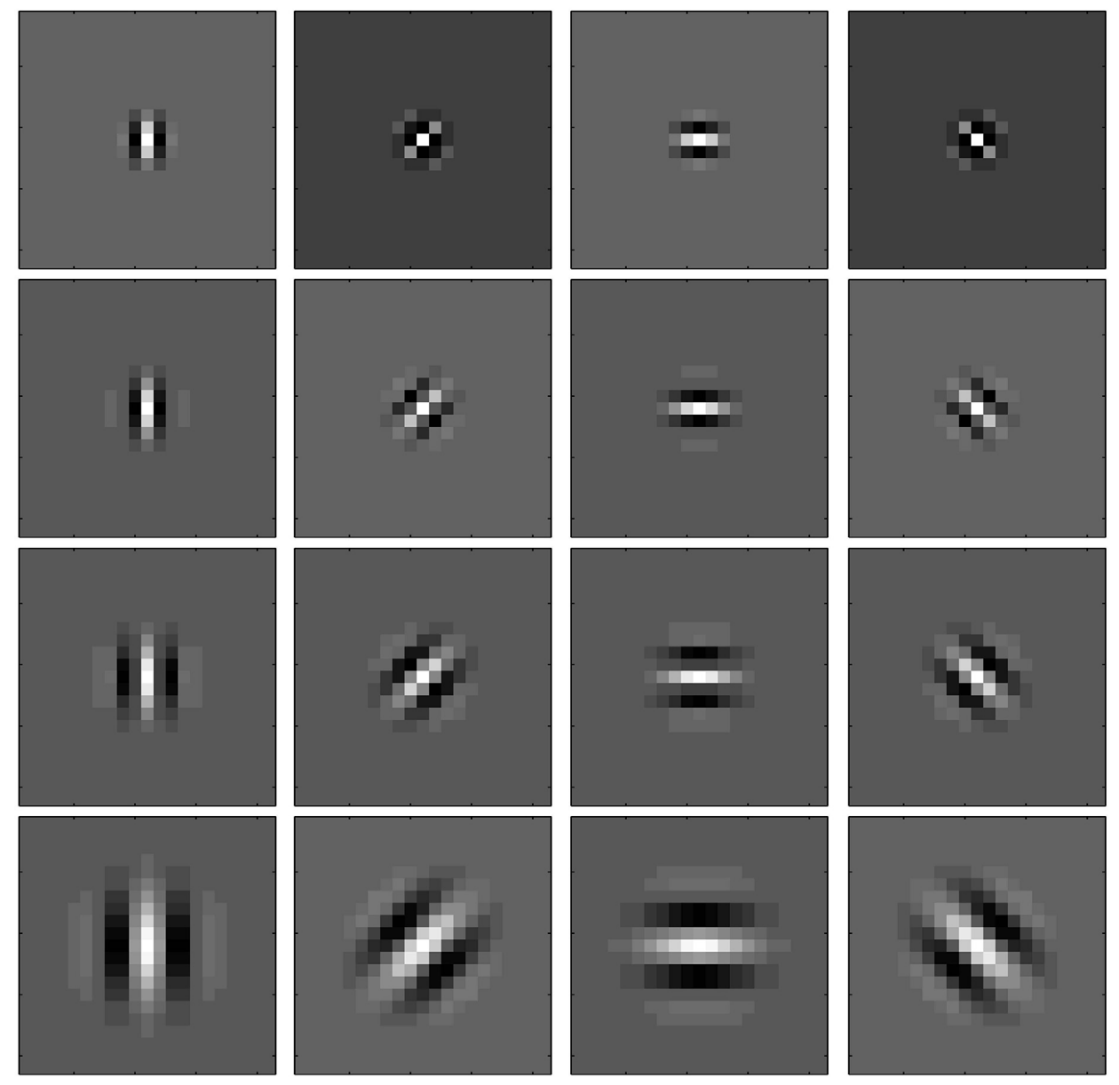
**Kernels used for the 4-orientation configuration**. Each row represents a different scale (from smaller to larger kernels). The maximum kernel value is 15 and the minimum is −7. Kernel size is 11 × 11 pixels.

**Figure 12:**
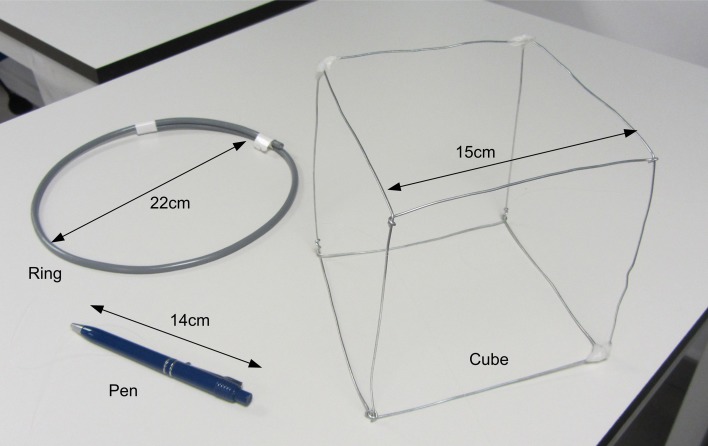
**Photograph of the three objects used to test the 3D reconstruction algorithm: a pen, a ring, and a cube**.

For the experimental evaluation of the 3D reconstruction, we analyzed the effect of several configurations of Gabor filters on the event matching algorithm B in order to compare them to algorithm A. For each configuration, we tested different numbers of orientation Gabor filters (from 2 to 8). All filters had always the same spatial scale, and we tested 4 different scales. Identical filters were applied to both retina outputs. Each row in Figure 11 shows an example of the kernels used in a configuration of 4 orientations (90, 45, 0, −45°), each configuration for a given spatial scale. In general, the different angles implemented in each case are uniformly distributed between 90 and −90°. This strategy was used to reconstruct in 3D the three objects shown in Figure 12: a 14 cm pen, a 22 cm diameter ring, and a 15 cm side metal wire cube structure.

#### Pen

A swinging pen of 14 cm length was moved in front of the two retinas for half a minute, with a number of approximately 100 Kevents generated by each retina. Table 1 summarizes the results of the 3D reconstruction, in terms of events. The column labeled “Orientations 0” corresponds to applying the matching algorithm directly to the retina pair outputs (algorithm A). When using Gabor filters (algorithm B), experiments with four different scales were conducted. For each scale, a different number of simultaneous filter orientations were tested, ranging from 2 to 8. In order to compare the performance of the stereo matching algorithm applied directly to the retinas (algorithm A, see section Event Matching) and applied to the outputs of the Gabor filters (algorithm B, see section Event Matching), the second row in Table 1 (*N*_*ev*_) shows the number of events processed by the algorithm in both cases. We show only the number of events coming originally from Retina 1, as they both have been configured to generate approximately the same number of events for a given stimulus.

**Table 1:** **Comparison of the 3D reconstruction results for the pen**.

		**Scale 1**							**Scale 2**						
Orientations	0	2	3	4	5	6	7	8	2	3	4	5	6	7	8
*N*_*ev*_	100	71	65	78	77	87	100	105	73	78	85	98	121	128	146
*N*_*m*_	28	15	14	15	14	16	17	18	15	15	16	18	22	24	27
Matching rate	28	21	21	19	19	18	17	17	21	20	19	18	18	19	19
Isolated events	2.9	5.6	6.4	5.4	5.8	5.1	4.5	4.1	5.1	4.9	4.7	4.1	3.0	2.6	2.1
*M*_*err*_	8.0	4.1	3.9	4.2	3.9	4.1	3.9	4.1	3.6	3.6	3.7	3.6	3.6	3.6	3.6
*N*_m-correct_	24.9	14	13	14	13	15	16	17	14	14	15	17	21	23	25
		**Scale 3**							**Scale 4**						
Orientations		2	3	4	5	6	7	8	2	3	4	5	6	7	8
*N*_*ev*_		74	80	87	106	131	154	169	77	79	85	99	129	145	170
*N*_*m*_		16	17	17	21	26	31	34	19	19	19	22	30	34	39
Matching rate		22	21	20	19	20	20	20	24	24	23	23	23	23	23
Isolated events		5.0	4.7	4.5	3.3	2.4	1.8	1.5	3.3	3.3	3.2	2.6	1.6	1.4	1.0
*M*_*err*_		5.2	5.2	5.2	5.1	4.9	4.9	5.0	8.3	8.3	8.3	8.3	8.2	8.1	7.8
*N*_m-correct_		14	15	15	19	24	29	32	17	17	17	20	27	31	36

The first column (0 orientations) presents the results obtained applying the matching algorithm to the retinas events (algorithm A, section Event Matching), while the rest of the columns are related to the pair-wise application of the matching algorithm to the outputs of the Gabor filters (algorithm B, section Event Matching), from Scale 1 (smaller kernels) to Scale 4 (larger kernels). For each scale, different numbers of orientations are considered (from 2 to 8), as indicated in the first row (Orientations). Second row (*N*_*ev*_) shows the number of events processed (in Kevents) by the matching algorithm in each case (i.e., the total number of events generated by all the filters). Third row (*N*_*m*_) presents the number of matched events (in Kevents) produced by the algorithm, while fourth row (*Matching Rate*) shows the ratio of matched events over the total number of events generated by the Gabor filters (*Matching Rate* = *100* · *N*_*m*_/N_*ev*_, in %). Fifth row (*Isolated events*) shows the ratio of isolated events over the total number of matched events (in %). Sixth row (*M*_*err*_) presents the ratio of wrongly matched events over the total number of matched events (in %). The last row (*N*_m-correct_) encapsulates the number of matched events with the ratio of isolated and wrongly matched events, presenting the number of correctly matched events (*N*_m-correct_ = *N*_*m*_ − $\left(\frac{Isolated\;events}{100}\cdot \;N_{m}\right)-\left(\frac{M_{err}}{100}\cdot \;N_{m}\right)$, in Kevents).

When the algorithm is applied directly to the output of the retinas, the number of matched pairs of events obtained is around 28 Kevents (28% of success rate). The third row in Table 1 (*N*_*m*_) shows the number of matched events for the different configurations of Gabors. If we calculate the percentage of success obtained by the algorithm for each configuration of filters in order to compare it with the 28% provided by the retinas alone, we obtain the values shown in the fourth row of Table 1 (*Matching Rate*).

**Figure 13:**
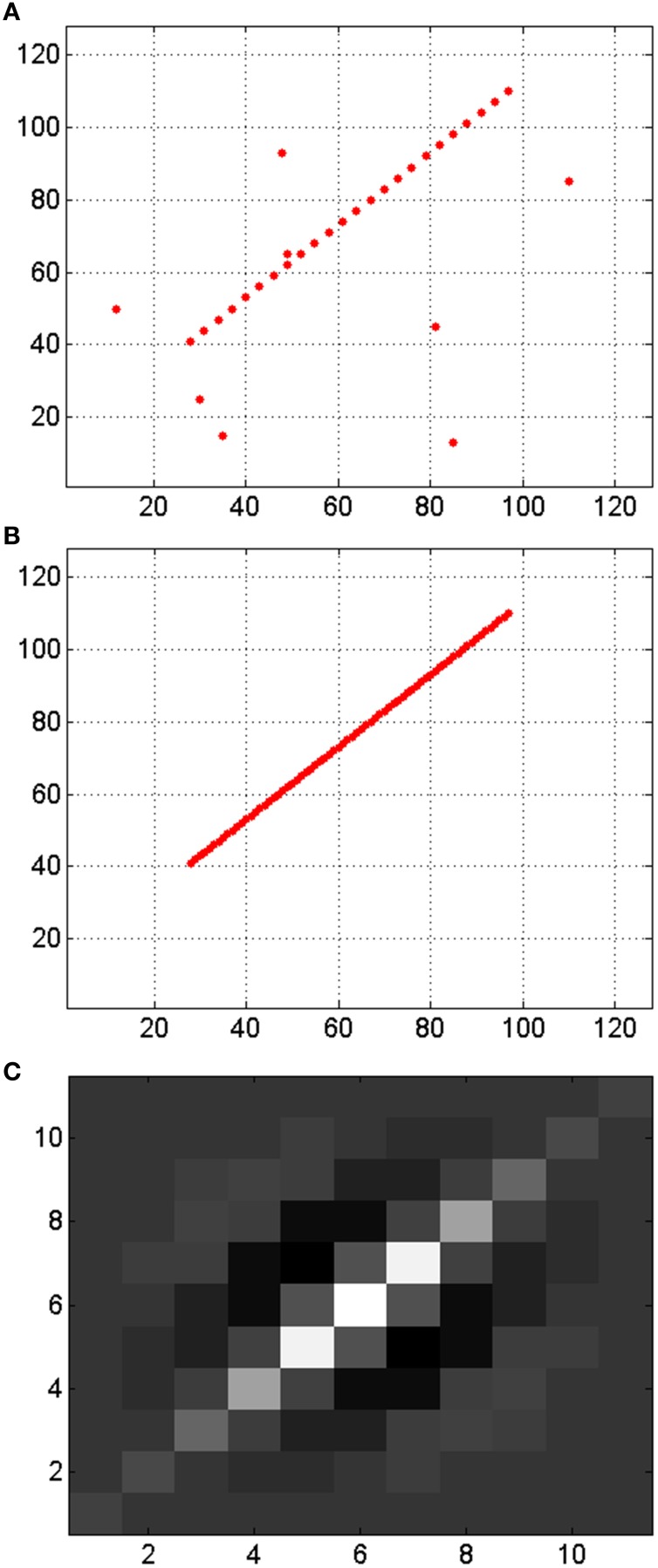
**Illustration of enhancing edges and noise reduction by a Gabor filter. (A)** Input events representing a discontinuous edge with noise. **(B)** Output events generated by the Gabor filter, with the reconstructed edge without noise. **(C)** Gabor kernel. All axes represent pixels, being the visual space in **(A,B)** 128 × 128 and the size of the kernel in **(C)** 11 × 11.

Although these results show that the matching rate of the algorithm is smaller when we use Gabor filters to extract information about the orientation of the edges that generated the events, we should consider that the performance of 3D reconstruction is determined by the total number of matched events, not the relative proportion. Note that the Gabor filters are capable of edge filling when detecting somewhat sparse or incomplete edges from the retina, thus enhancing edges and providing more events for these edges. Figure 13 shows an example where a weak edge (in Figure 13A) produced by a retina together with noise events is filled by a Gabor filter (with the kernel shown in Figure 13C) producing the enhanced noise-less edge in Figure 13B, and increasing the number of edge events from 24 to 70 while removing all retina-noise events. The more matched events, the better 3D reconstruction. For that reason, we consider that a bank of 8 Gabor filters with kernels of scale 4 gives the best result, with more than 39 Kevents that can be used to reconstruct the 3D sequence, using 100 Kevents generated by the retinas. This application of Gabor filters for edges filling was first demonstrated in (Lindenbaum et al., 1994), and has also been used for fingerprint image enhancement (Hong et al., 1998; Greenberg et al., 2002).

Another parameter that can be used to measure the quality of the 3D reconstruction is the proportion of “isolated” events in the matched sequence. We define an isolated event as an event which is not correlated to any other event in a certain spatio-temporal window, meaning that no other event has been generated in its neighbor region within a limited time range. A non-isolated event (an event generated by an edge of the object) will be correlated to some other events generated by the same edge, which will be close in space and time. Note that these isolated matched events correspond to false matches. These false matches can be produced when an event in one retina is matched by mistake with a noise event in the other retina, or when two or more events that happen very simultaneously in 3D space are cross-matched by the matching algorithm. With this definition of isolated events, the 28 Kevents that were matched for the retinas without any filtering were used to reconstruct the 3D coordinates of these events, resulting in only 2.93% of isolated events. After the application of the same methodology to all the Gabor filters configurations, the results in the fifth row in Table 1 (*Isolated events*) are obtained. These results show that several configurations of Gabor filters give a smaller proportion of isolated events.

In order to remove the retina-noise events, it is also possible to insert a noise removal block directly at the output of the retina (jAER, 2007). However, this introduces a small extra latency before the events can be processed, thus limiting event-driven stereo vision for very high speed applications (although it can be a good solution when timing restrictions are not too critical). The effect of Gabor filters on noise events is also illustrated in Figure 13, where all the events that were not part of an edge with the appropriate orientation are removed by the filter. However, it is possible that some noise events add their contributions together producing noise events at the output of the Gabor filters. Two different things can happen with these events: (1) the stereo matching algorithm does not find a corresponding event in the other retina; (2) there is a single event which satisfies all restrictions, so a 3D point will be reconstructed from a noise event, producing a wrongly matched event, as is described in the next paragraph.

**Figure 14:**
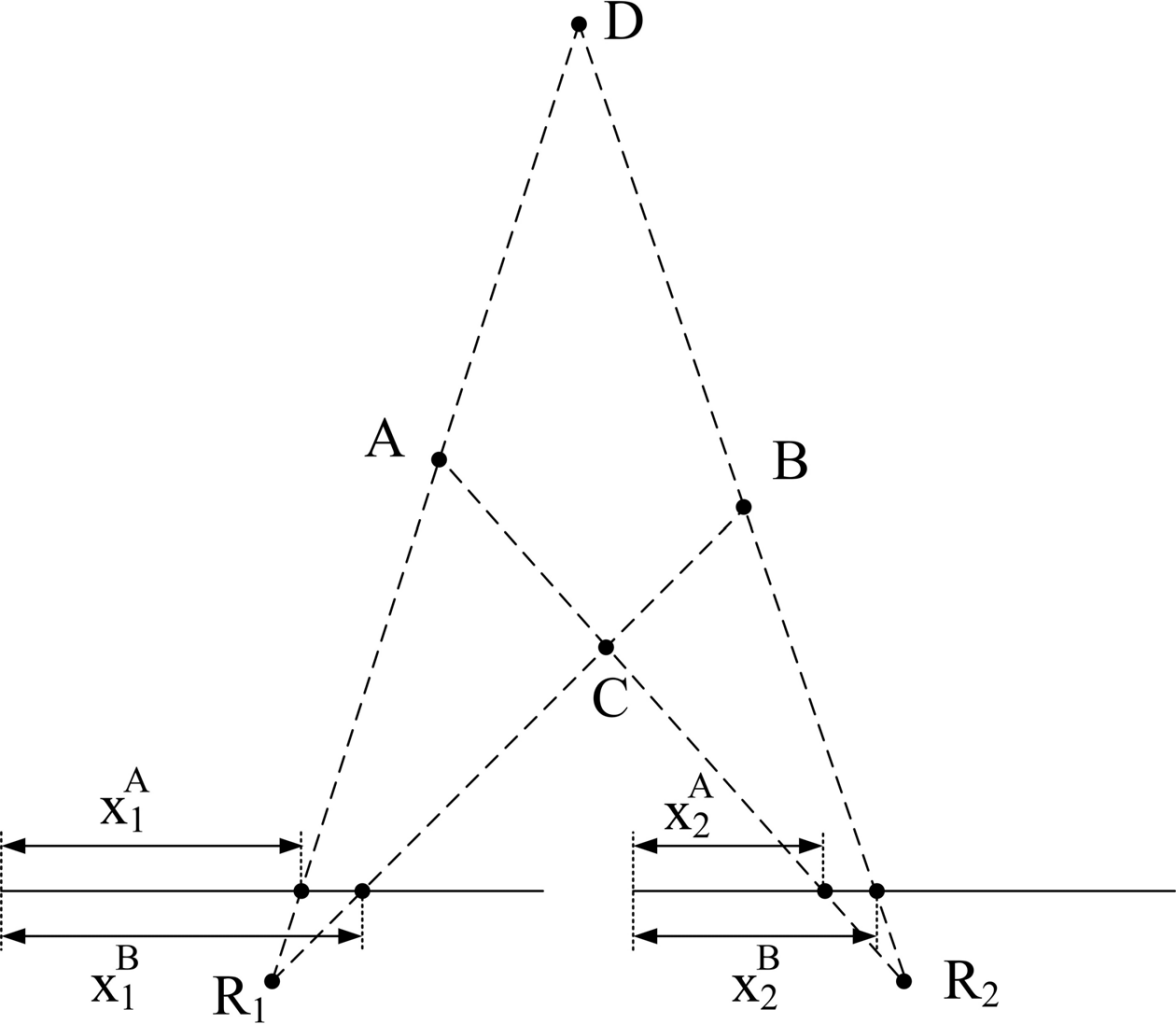
**Illustration of matching errors**.

**Figure 15:**
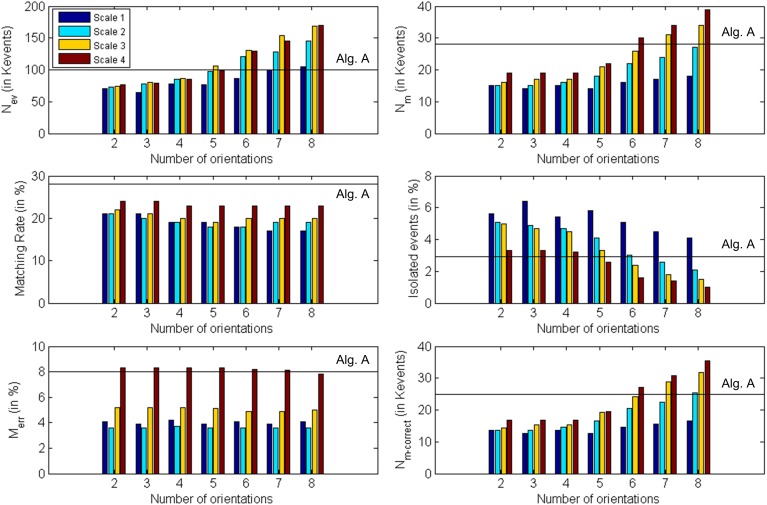
**Graphical representation of Table 1**. Each subplot corresponds to a different row of the table, showing the obtained values for each number of orientations and scale. The black horizontal lines indicate the values obtained using algorithm A (0 orientations).

Although the object used in this first example is very simple, we must consider the possibility that the algorithm matches wrongly some events. In particular, if we think about a wide object we can have events generated simultaneously by two far edges: the left and the right one. Therefore, it can happen that an event corresponding to the left edge in Retina 1 does not have a proper partner in Retina 2, but another event generated by the right edge in Retina 2 might satisfy all the restrictions imposed by the matching algorithm. Figure 14 illustrates the mechanism that produces this error. Let us assume that the 3D object has its left and right edges located at positions A and B in 3D space. Locations A and B produce events at *x*^*A*^_1_ and *x*^*B*^_1_ in Retina 1, and at *x*^*A*^_2_ and *x*^*B*^_2_ in Retina 2. These events are the projections onto the focal points *R*_1_ and *R*_2_ of both retinas, activating pixels (*x*^*j*^_*i*_, *y*^*j*^_*i*_), with *i* = 1,2 and *j* = *A*,*B*. Therefore, an event generated in Retina 1 with coordinates (*x*^*A*^_1_, *y*^*A*^_1_) should match another event generated in Retina 2 with coordinates (*x*^*A*^_2_, *y*^*A*^_2_). However, note that in Figure 13, an edge at position D is captured by Retina 1 at the same pixel that an edge at A, and in Retina 2 they would be on the same epipolar lines. The same happens for edges at positions B and C. Consequently, it can happen that no event is produced in Retina 2 at coordinate (*x*^*A*^_2_, *y*^*A*^_2_) at the same time, but another event with coordinates (*x*^*B*^_2_, *y*^*B*^_2_) is generated within a short time range by the opposite simultaneously moving edge, being those coordinates in the same epipolar line. In that case, the algorithm might match (*x*^*A*^_1_, *y*^*A*^_1_) with (*x*^*B*^_2_, *y*^*B*^_2_), reconstructing a wrong 3D point in coordinate D. The opposite combination would produce a wrong 3D event in point C. This effect could produce false edges in the 3D reconstruction, especially when processing more complex objects. However, the introduction of the Gabor filters to extract the orientation of the edges will reduce the possibility of matching wrong pairs of events. In order to measure the proportion of wrongly matched events, we consider that all the good pairs of events will follow certain patterns of disparity, so all the events which are close in time will be included within a certain range of disparity values. Calculating continuously the mean and standard deviation of the distribution of disparities, we define the range of acceptable values, and we identify as wrongly matched all those events whose disparity is outside that range. Using this method, we calculate the proportion of wrongly matched events and present it (in %) in the sixth row of Table 1 (*M*_*err*_). Finally, the last row presents the number of correctly matched events, subtracting both the isolated and wrongly matched events from the total number of matched events: *N*_m−correct_ = *N*_*m*_ − $\left(\frac{\text{Isolated events}}{100}\cdot \;N_{m}\right)\;-\;\left(\frac{M_{\text{err}}}{100}\cdot \;N_{m}\right)$. All these results are presented graphically in Figure 15, where the colored vertical bars represent the results obtained applying algorithm B with different number of orientations and scales, while the black horizontal lines indicate the values obtained using algorithm A (no Gabor filters). From this figure, we decide that the best case is 8 orientations and Scale 4, as it provides the largest number of correctly matched events. However, it could also be argued that 8 orientations and Scale 3 gives a smaller number of wrongly matched events, but in that case the number of correctly matched events is also smaller.

**Figure 16:**
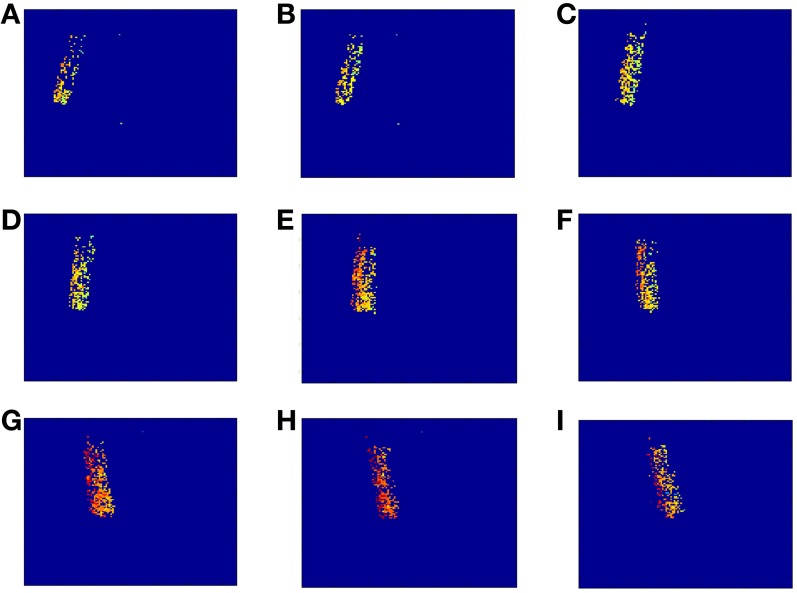
**Sequence of disparity maps**. They were reconstructed with *T*_frame_ = 50 ms and they correspond to the movement of the swinging pen (from **A**–**I**). The disparity scale goes from dark blue to red to encode events from far to near.

Using the sequence of matched events provided by the algorithm in the best case (8 orientations, Scale 4), we computed the disparity map. The underlying reasons why this configuration provides the best result are: (a) Scale 4 matches better the scale of the object edges in this particular case, and (b) given the object geometry and its tilting in time, a relatively fine orientation angle detection was required. If we compare this case with the results obtained applying algorithm A without Gabor filters (first column in Table 1), we observe an increase of 39% in the number of matched events, while the proportions of isolated events and wrongly matched pairs have decreased by 65 and 2.5%, respectively. Moreover, the number of correctly matched events has increased by 44%. In order to compute the disparity map, we calculated the euclidean distance between both pixels in each pair of events (from Retina 1 and Retina 2). This measurement is inversely proportional to the distance between the represented object and the retinas, as further objects produce a small disparity and closer objects produce a large disparity value. Figure 16 shows 9 consecutive frames of the obtained disparity sequence, with a frame time of 50 ms. The disparity scale goes from dark blue to red to encode events from far to close.

**Figure 17:**
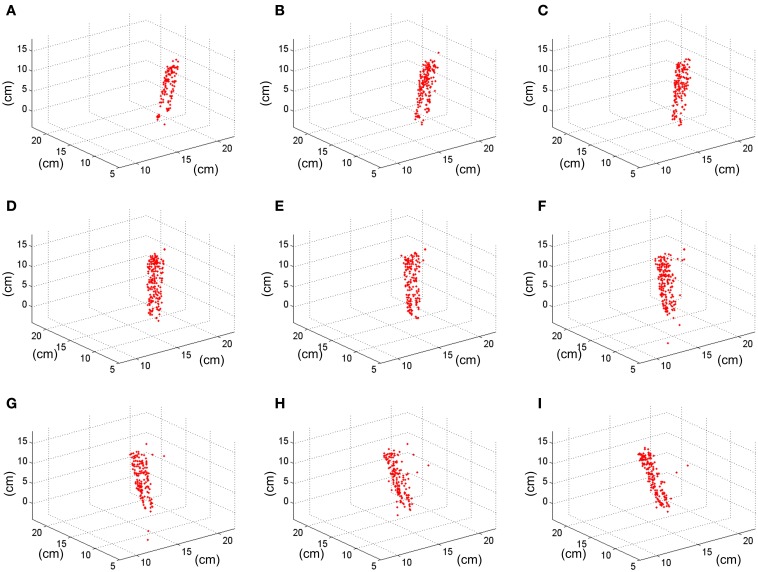
**Result of the 3D reconstruction of the swinging pen recording**. Each plot (from **A**–**I**) corresponds to a 50 ms-frame representation of the 3D coordinates of the matched events.

Applying the method described in section 3D Reconstruction, the 3 dimensional coordinates of the matched events are calculated. Figure 17 shows 9 consecutive frames of the resultant 3D reconstruction, with a frame time of 50 ms. The shape of the pen is clearly represented as it moves around 3D space. Using this sequence, we measured manually the approximate length of the pen by calculating the distance between the 3D coordinates of pairs of events located in the upper and lower limits of the pen, respectively. This gave an average length of 14.85 cm, being the real length 14 cm, which means an error of 0.85 cm. For an approximate distance to the retinas of 1 m, the maximum error predicted in Figure 10 would be below 1.5%, resulting in 1.5 cm. Therefore, we can see that the 0.85 cm error is smaller than the maximum predicted by Figure 10.

#### Ring

**Figure 18:**
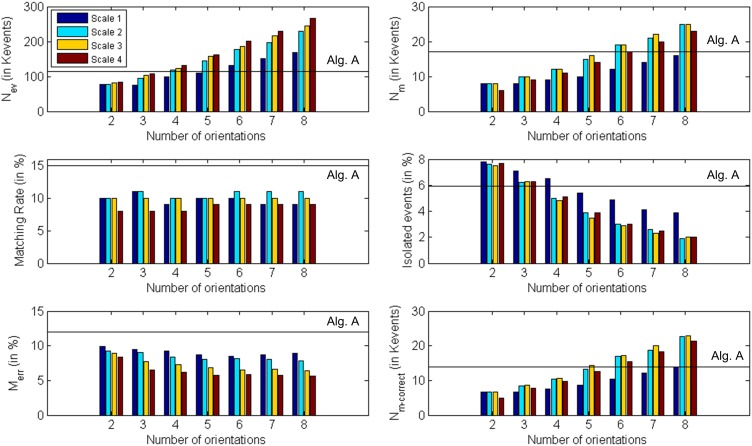
**Graphical representation of Table 2**. Each subplot corresponds to a different row of the table, showing the obtained values for each number of orientations and scale. The black horizontal lines indicate the values obtained using algorithm A (0 orientations).

**Figure 19:**
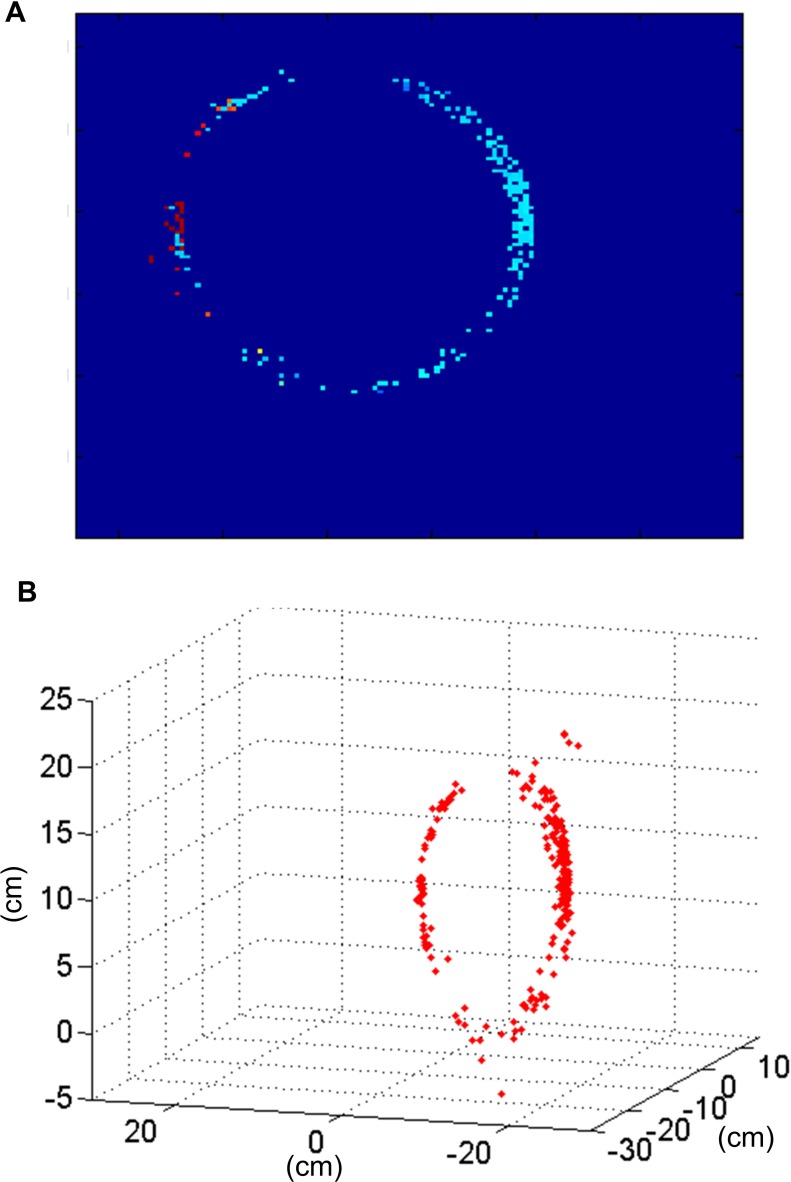
**Results obtained for the rotating ring. (A)** Disparity map reconstructed with *T*_frame_ = 50 ms corresponding to the rotation of the ring. **(B)** Result of the 3D reconstruction of the same frame of the ring recording.

A ring with a diameter of 22 cm was rotating slowly in front of the two retinas for half a minute, with a number of approximately 115 Kevents generated by each retina. As in the previous example, the matching algorithm was applied both to the events generated by the retinas (see section Event Matching, algorithm A) and to the events generated by the Gabor filters (see section Event Matching, algorithm B), in order to compare both methods. Table 2 shows all the results for all the configurations of Gabor filters (from 2 to 8 orientations, with scales 1–4). All these results are presented graphically in Figure 18, where the colored vertical bars represent the results obtained applying algorithm B with different number of orientations and scales, while the black horizontal lines indicate the values obtained using algorithm A (no Gabor filters). We can see in the table how the largest number of matched events (25 K) is obtained for 8 orientations and both scales 2 and 3. Although the ratio of noise events is very similar for both of them (1.9% for Scale 2 and 2.0% for Scale 3), Scale 3 provides a smaller ratio of wrongly matched events (7.8% for Scale 2 and 6.4% for Scale 3). Therefore, we conclude that the best performance is found with 8 orientations and Scale 3, as it is more appropriate to the geometry of the object. If we compare this case with the results obtained applying algorithm A without Gabor filters (first column in Table 2), we observe an increase of 47% in the number of matched events, while the proportions of isolated events and wrongly matched pairs have decreased by 66 and 46%, respectively. Therefore, the number of correctly matched events has increased by 64%. A frame reconstruction of the disparity map and the 3D sequence are shown in Figure 19.

**Table 2:** **Comparison of the 3D reconstruction results for the ring**.

		**Scale 1**							**Scale 2**						
Orientations	0	2	3	4	5	6	7	8	2	3	4	5	6	7	8
*N*_*ev*_	115	78	75	100	109	131	151	168	78	95	119	143	177	197	229
*N*_*m*_	17	8	8	9	10	12	14	16	8	10	12	15	19	21	25
Matching rate	15	10	11	9	10	10	9	9	10	11	10	10	11	11	11
Isolated events	5.9	7.8	7.1	6.5	5.4	4.9	4.1	3.9	7.6	6.2	5.0	3.9	3.0	2.6	1.9
*M*_*err*_	12.0	9.9	9.5	9.3	8.7	8.5	8.7	8.9	9.3	9.0	8.4	8.1	8.2	8.0	7.8
*N*_m-correct_	14	7	7	8	9	10	12	14	7	8	10	13	17	19	23
		**Scale 3**							**Scale 4**						
Orientations		2	3	4	5	6	7	8	2	3	4	5	6	7	8
*N*_*ev*_		82	103	122	157	185	217	245	83	107	131	161	201	229	266
*N*_*m*_		8	10	12	16	19	22	25	6	9	11	14	17	20	23
Matching rate		9	10	10	10	10	10	10	8	8	8	9	9	9	9
Isolated events		7.5	6.3	4.8	3.5	2.9	2.3	2.0	7.7	6.3	5.1	3.9	3.0	2.5	2.0
*M*_*err*_		8.9	7.7	7.3	6.8	6.5	6.6	6.4	8.4	6.5	6.2	5.7	5.9	5.8	5.6
*N*_m-correct_		7	9	11	14	17	20	23	5	8	10	13	15	18	21

The meaning of the columns and rows is as in Table 1.

The diameter of the reconstructed ring was measured manually by selecting pairs of events with the largest possible separation. This gave an average diameter of 21.40 cm, which implies a reconstruction error of 0.6 cm. This error is also smaller than the maximum predicted in Figure 10.

#### Cube

**Figure 20:**
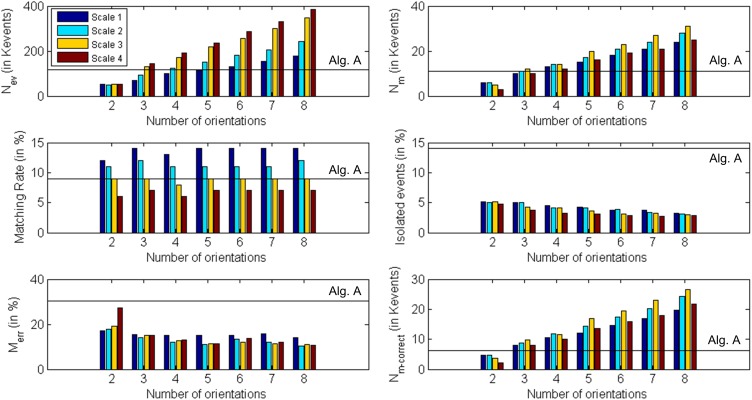
**Graphical representation of Table 3**. Each subplot corresponds to a different row of the table, showing the obtained values for each number of orientations and scale. The black horizontal lines indicate the values obtained using algorithm A (0 orientations).

Finally, a cube with an edge length of 15 cm was rotating in front of the retinas, with a number of approximately 118 Kevents generated by each retina in approximately 20 s. The same procedure performed in previous examples was repeated, obtaining the results shown in Table 3. All these results are presented graphically in Figure 20, where the colored vertical bars represent the results obtained applying algorithm B with different number of orientations and scales, while the black horizontal lines indicate the values obtained using algorithm A (no Gabor filters). In this case, the largest number of matched events (31 K) is given by 8 orientations and Scale 3, while both the ratio of isolated events and the ratio of wrongly matched events are very similar for the four different scales with 8 orientations (around 3% noise and 10.9% wrong matches). Therefore, the best performance is given by 8 orientations and Scale 3. If we compare this case with the results obtained applying algorithm A without Gabor filters (first column in Table 3), we observe an increase of 181% in the number of matched events, while the proportions of isolated events and wrongly matched pairs have decreased by 78 and 46%, respectively. The number of correctly matched events has increased by 350%.

**Table 3:** **Comparison of the 3D reconstruction results for the cube**.

		**Scale 1**							**Scale 2**						
Orientations	0	2	3	4	5	6	7	8	2	3	4	5	6	7	8
*N*_*ev*_	118	54	68	100	112	132	153	178	50	93	125	152	183	205	243
*N*_*m*_	11	6	10	13	15	18	21	24	6	11	14	17	21	24	28
Matching rate	9	12	14	13	14	14	14	14	11	12	11	11	11	11	12
Isolated events	14.0	5.2	5.0	4.5	4.3	3.8	3.7	3.3	5.0	5.0	4.1	4.1	3.9	3.4	3.1
*M*_*err*_	20.3	17.0	15.5	15.1	15.0	15.1	15.8	14.1	17.9	14.2	11.9	11.1	13.3	12.0	10.3
*N*_m-correct_	6	5	8	10	12	15	17	20	5	9	12	14	17	20	24
		**Scale 3**							**Scale 4**						
Orientations		2	3	4	5	6	7	8	2	3	4	5	6	7	8
*N*_*ev*_		54	130	170	219	256	300	346	51	145	190	235	285	329	386
*N*_*m*_		5	12	14	20	23	27	31	3	10	12	16	19	21	25
Matching rate		9	9	8	9	9	9	9	6	7	6	7	7	7	7
Isolated events		5.2	4.2	4.1	3.6	3.1	3.2	3.0	4.8	3.7	3.2	3.1	2.9	2.7	2.8
*M*_*err*_		19.0	15.1	12.7	11.3	11.9	11.2	10.9	27.4	15.0	12.9	11.4	13.7	12.2	10.7
*N*_m-correct_		4	10	12	17	20	23	27	2	8	10	14	16	18	22

The meaning of the columns and rows is as in Table 1.

**Figure 21:**
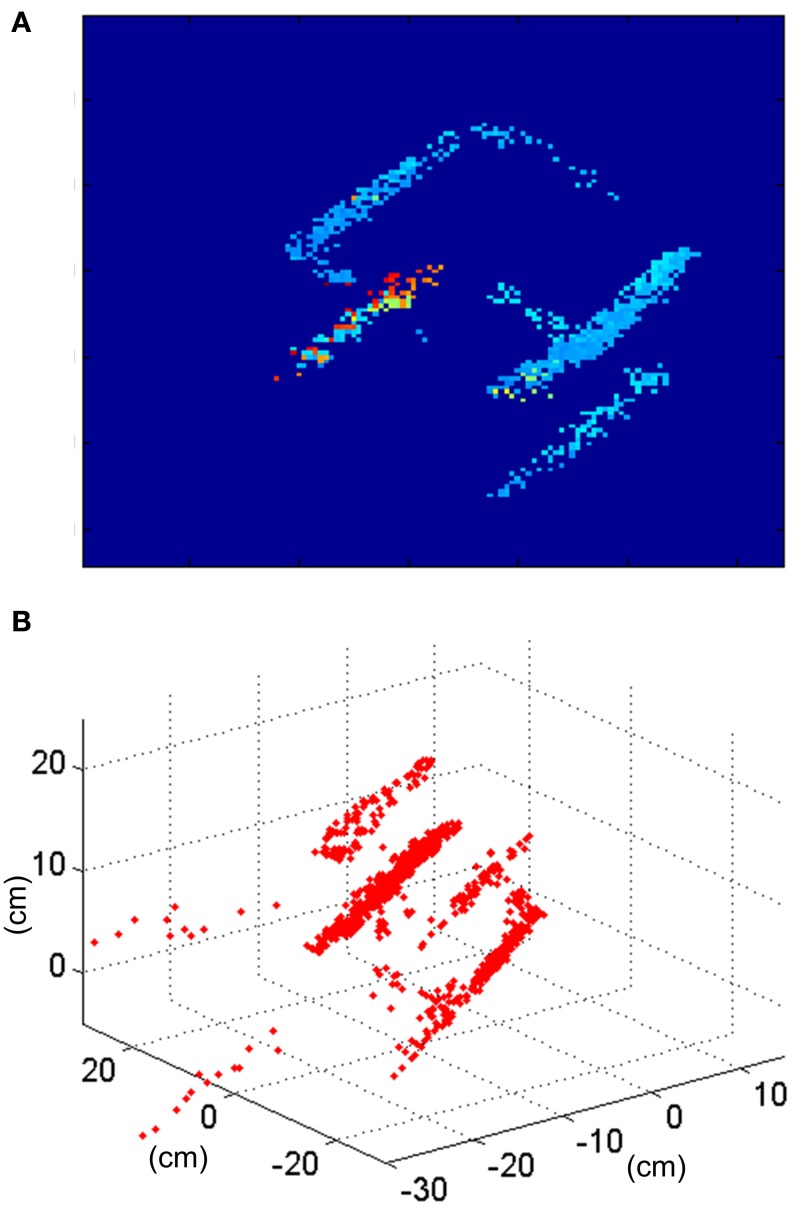
**Results obtained for the cube. (A)** Disparity map reconstructed with *T*_frame_ = 50 ms corresponding to the rotation of the cube. **(B)** Result of the 3D reconstruction of the same frame of the cube recording.

A reconstruction of the disparity map and the 3D sequence is shown in Figure 21. The ratio of wrongly matched events is much larger than on the ring example (about twice as much). That is because this object has many parallel edges, increasing the number of events in the same epipolar line which are candidates to be matched and which the orientation filters do not discriminate. While Figure 14 shows a situation where 2 different positions in 3D space (A and B) can generate events that could be wrongly matched, in this case we could find at least 4 different positions in 3D space (as we have 4 parallel edges) with the same properties.

The edge length of the reconstructed 3D cube was measured manually on the reconstructed events, giving an average length of 16.48 cm, which implies a reconstruction error of 1.48 cm. This error is smaller than the maximum predicted in Figure 10.

## Conclusion

This paper analyzes different strategies to improve 3D stereo reconstruction in event-based vision systems. First of all, a comparison between stereo calibration methods showed that by using a calibration object with LEDs placed in known locations and measuring their corresponding 2D projections with sub-pixel resolution, we can extract the geometric parameters of the stereo setup. This method was tested by reconstructing the known coordinates of the calibration object, giving a mean error comparable to the size of each LED.

Event matching algorithms have been proposed for stereo reconstruction, taking advantage of the precise timing information provided by DVS sensors. In this work, we have explored the benefits of using Gabor filters to extract the orientation of the object edges and match events from pair wise filters directly. This imposes the restriction that the distance from the stereo cameras to the objects must be much larger than the focal length of the lenses, so that edge orientations appear similar in both cameras. By analyzing different numbers of filters with several spatial scales, we have shown that we can increase the number of reconstructed events for a given sequence, reducing the number of both noise events and wrong matches at the same time. This improvement has been validated by reconstructing in 3D three different objects. The size of these objects was estimated from the 3D reconstruction, with an error smaller than theoretically predicted by the method (1.5%).

### Conflict of interest statement

The authors declare that the research was conducted in the absence of any commercial or financial relationships that could be construed as a potential conflict of interest.

## Footnotes

^1^There is, however, a “pathological” exception: a very thin and long object, perfectly centred between the two retinas, having its long dimensión perpendicular to the retina planes, may produce different angles at both retinas.
