# Supplementary material for: On the use of orientation filters for 3D reconstruction in event-driven stereo vision
Source: Front Neurosci. 2014 Mar 31;8:48. doi: 10.3389/fnins.2014.00048 (PMC3978326; doi:10.3389/fnins.2014.00048)
Supplement: Supplementary file 1 [file Presentation1.PDF]

Appendix A. Calculation of projection matrix P

The pinhole camera model is used to describe the mathematical relationship between the coordinates of a 3D point and its projection onto the image (Hartley and Zisserman, 2003). Under these assumptions, the relationship between $\widetilde{m}$ and $\widetilde{M}$ is given by:

$$s \cdot \widetilde{m} = A \cdot [R \quad T] \cdot \widetilde{M} \qquad \text{(A.1)}$$

where s is an arbitrary scale factor, A is the camera intrinsic matrix, and R, T are the extrinsic parameters (rotation and translation matrices, respectively). Camera calibration is the procedure to calculate all these intrinsic and extrinsic parameters in order to be able to relate 3D metric information and 2D projection points.

The projection matrix for camera i can be defined as $P_i = A_i \cdot [R_i \quad T_i]$ (being P_i a $3x4$ matrix). Considering scale factor $s = 1$, equation (A.1) can be rewritten as:

$$\widetilde{m} = P_i \cdot \widetilde{M} \qquad \text{(A.2)}$$

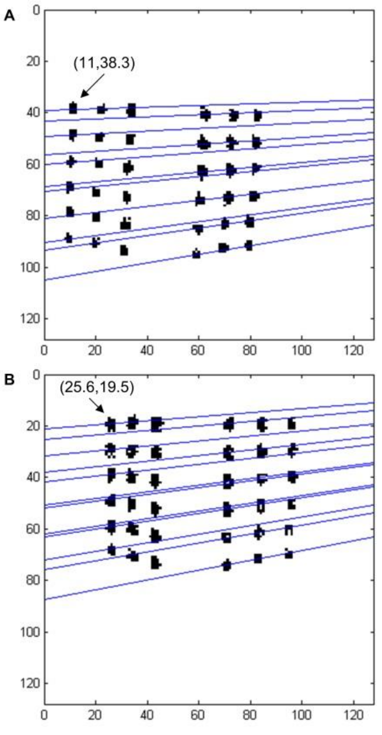

FIGURE A.1. 2D images representing the activity recorded by both retinas during calibration after binarization. Each cluster of pixels corresponds to a blinking LED. Some epipolar lines are shown. The disparity of the upper left LED is 23.8 pixels, and the mean disparity for all the LEDs is 24.55 pixels.

Therefore, knowing the projection matrices of the different cameras in a vision system can be enough to extract the coordinates of the 3D points in space from their corresponding 2D projections (shown in **Figure A.1**), with no need to obtain all the specific intrinsic and extrinsic parameters. This represents an important simplification of the calibration procedure.

After calculating $\widetilde{m}_1^j$ and $\widetilde{m}_2^j$ $(j = 1, \dots 36)$ and knowing $\widetilde{M}^j$, we can apply any algorithm that was developed for traditional frame-based computer vision (Longuet-Higgins, 1981) to extract P_1 and P_2. For the algorithm used in this work, equation (A.2) can be rewritten as:

$$\widetilde{m} \times P\widetilde{M} = 0 \qquad \text{(A.3)}$$

The terms in equation (A.3) can be rearranged to obtain a new equation in the form $Ab = 0$:

$$\begin{pmatrix} 0_{1x4} & -\widetilde{M}^T & y\widetilde{M}^T \\ \widetilde{M}^T & 0_{1x4} & -x\widetilde{M}^T \\ -y\widetilde{M}^T & x\widetilde{M}^T & 0_{1x4} \end{pmatrix} \begin{pmatrix} P_1^T \\ P_2^T \\ P_3^T \end{pmatrix} = 0_{3x1} \qquad \text{(A.4)}$$

where P_i^T is the $i - th$ row of the transposed projection matrix. Given that only two rows of matrix A are linearly independent, if we have n different calibration points, we can write:

$$\begin{pmatrix} 0_{1x4} & -\widetilde{M}_1^T & y_1\widetilde{M}_1^T \\ \widetilde{M}_1^T & 0_{1x4} & -x_1\widetilde{M}_1^T \\ \vdots & \vdots & \vdots \\ 0_{1x4} & -\widetilde{M}_n^T & y_n\widetilde{M}_n^T \\ \widetilde{M}_n^T & 0_{1x4} & -x_n\widetilde{M}_n^T \end{pmatrix} \begin{pmatrix} P_1^T \\ P_2^T \\ P_3^T \end{pmatrix} = 0_{2nx1} \qquad \text{(A.5)}$$

where $\widetilde{M}_i^T$ represents the 3D coordinates of the $i - th$ calibration point, and (x_i, y_i) the camera 2D coordinates of the $i - th$ calibration point. This can be solved as a linear least squares minimization problem, giving matrix P as a solution. One equivalent system must be solved for each camera.

Appendix B. Calculation of reconstructed 3D coordinates

In order to solve equations (3), we rearrange terms to obtain a single equation in the form $Ab = 0$ (Hartley and Zisserman, 2003):

$$\begin{pmatrix} -P_{i,2} + y_i P_{i,3} \\ P_{i,1} - x_i P_{i,3} \\ -y_i P_{i,1} + x_i P_{i,2} \end{pmatrix} \widetilde{M} = 0_{3x1} \qquad \text{(B.1)}$$

where $P_{i,j}$ represents the $j - th$ row of the projection matrix corresponding to retina i $(i = 1,2)$. Given that only two rows of this matrix are linearly independent, we can write the following system:

$$\begin{pmatrix} -P_{1,2} + y_1 P_{1,3} \\ P_{1,1} - x_1 P_{1,3} \\ -P_{2,2} + y_2 P_{2,3} \\ P_{2,1} - x_2 P_{2,3} \end{pmatrix} \widetilde{M} = 0_{4x1} \qquad \text{(B.2)}$$

This can be solved as a linear least squares minimization problem, giving the final 3D coordinates $M = [X\ Y\ Z]^T$ as a solution.
